# Can thyroidectomy be considered safe in obese patients? A retrospective cohort study

**DOI:** 10.1186/s12893-020-00939-w

**Published:** 2020-11-07

**Authors:** Gian Luigi Canu, Fabio Medas, Federico Cappellacci, Michele Guido Podda, Giorgio Romano, Enrico Erdas, Pietro Giorgio Calò

**Affiliations:** 1 Department of Surgical Sciences, University of Cagliari, “Policlinico Universitario Duilio Casula”, 09042 Monserrato, CA Italy; 2 Department of Surgical, Oncological and Oral Sciences, Section of General and Urgent Surgery, University of Palermo, 90127 Palermo, Italy

**Keywords:** Thyroidectomy, Endocrine surgery, Obesity, Body mass index, BMI, Complications

## Abstract

**Background:**

Obesity is a growing public health concern in most western countries. More and more patients with high body mass index (BMI) are undergoing surgical procedures of all kinds and, in this context, obese patients are undergoing thyroid surgery more than ever before.

The aim of the present study was to evaluate whether thyroidectomy can be considered safe in obese patients.

**Methods:**

Patients undergoing thyroidectomy in our Unit between January 2014 and December 2018 were retrospectively analysed.

Patients were divided into two groups: those with BMI < 30 kg/m^2^ were included in Group A, while those with BMI ≥ 30 kg/m^2^ in Group B. Univariate analysis was performed to compare these two groups.

Moreover, multivariate analyses were performed to evaluate whether the BMI value (considered in this case as a continuous variable) had a significant role in the development of each individual postoperative complication.

**Results:**

A total of 813 patients were included in this study: 31 (3.81%) were underweight, 361 (44.40%) normal-weight, 286 (35.18%) overweight, 94 (11.57%) obese and 41 (5.04%) morbidly obese. Six hundred and seventy-eight patients were included in Group A and 135 in Group B.

At univariate analysis, the comparison between the two groups, in terms of operative time and thyroid weight resulted in statistically significant results (*P* = 0.001, *P* = 0.008; respectively). These features were significantly higher in Group B than in Group A. About postoperative stay and complications, no statistically significant difference was found between the two groups.

At multivariate analyses, only the development of cervical haematoma was statistically significantly correlated to the BMI value. Patients with high BMI had a lower risk of cervical haematoma (*P* = 0.045, OR 0.797, 95% CI 0.638–0.995).

**Conclusions:**

This study showed that obesity, in the field of thyroid surgery, is not associated with any increase of postoperative complications. Thus, it is possible to conclude that thyroidectomy can be performed safely in obese patients. Our result about operative times had no clinical significance.

## Background

Obesity is a growing public health concern in most western countries. The World Health Organization (WHO), in 2016, stated that obesity had nearly tripled since 1975 worldwide with more than 1.9 billion adults overweight, among whom 650 million obese [1].

Elevated body mass index (BMI) represents a major risk factor for several comorbidities, such as diabetes mellitus, musculoskeletal disorders, cardiovascular disease and some types of cancers, including thyroid carcinoma [2–5]. These comorbidities increase medical costs, which can be particularly burdensome considering the overall increase in life expectancy [2].

More and more patients with high BMI are undergoing surgical procedures of all kinds and, in this context, obese patients are undergoing thyroid surgery more than ever before [6–9]. For this reason, there is a growing interest in understanding the impact of elevated BMI on surgical outcomes.

Obesity is considered by many surgeons, including endocrine surgeons, as a condition associated with worse surgical outcomes and postoperative complications.

However, in the literature, substantial controversy exists about a correlation between high BMI and postoperative morbidity and mortality in numerous surgical fields. Some authors, for example, observed that elevated BMI increases morbidity in elective spine surgery and laparoscopic colorectal surgery, while, on the other hand, in a large review of 101,078 patients who underwent emergency abdominal operations, obesity has even been described as protective against mortality [6–8].

About thyroid surgery and obesity, the literature is limited. Moreover, among existing studies, most of them include a limited number of patients, while those with the largest populations do not evaluate recurrent laryngeal nerve (RLN) injury and hypoparathyroidism, which are the two main complications of thyroidectomy [4, 5, 10–18].

The aim of the present study was to evaluate whether thyroidectomy can be considered safe in obese patients.

## Methods

### Study design

In this retrospective cohort study we considered patients undergoing thyroidectomy between January 2014 and December 2018 in our Unit of General and Endocrine Surgery (University of Cagliari).

Patients were identified from a prospectively maintained institutional database.

Only patients who underwent conventional open total thyroidectomy, performed by the two most skilled endocrine surgeons of our Unit, were included. We excluded from this study patients simultaneously submitted to parathyroidectomy, parathyroid autotransplantation or lateral and/or central lymph node dissection and those with incomplete data.

BMI values were stratified according to the standardized categories set by the World Health Organization: < 18.5 kg/m^2^ (underweight), 18.5–24.9 kg/m^2^ (normal-weight), 25–29.9 kg/m^2^ (overweight), 30–34.9 kg/m^2^ (obese), ≥ 35 kg/m^2^ (morbidly obese) [19].

Enrolled patients were divided into two groups: those with BMI < 30 kg/m^2^ were included in Group A, while those with BMI ≥ 30 kg/m^2^ in Group B.

Demographic data, BMI, histological findings, surgical outcomes (operative time, postoperative stay) and postoperative complications (hypoparathyroidism, recurrent laryngeal nerve injury, cervical haematoma, wound infection) were analysed.

### Endpoints

The primary endpoint was the occurrence of postoperative complications, while secondary endpoints were operative time and postoperative stay.

### Surgical procedure

All patients were euthyroid at the time of surgery. Parathyroid glands and recurrent laryngeal nerves were systematically searched and identified. In order to facilitate nerve identification and to confirm its functional integrity, intraoperative nerve monitoring (IONM) was often utilized. Energy-based devices were routinely used to achieve haemostasis. The duration of the operation was estimated from skin incision to skin closure (in minutes).

### Postoperative management and classification of complications

Postoperative fibrolaryngoscopy was performed to assess vocal cord mobility in case of suspected recurrent laryngeal nerve injury.

Serum calcium and PTH values were assayed pre- and postoperatively. Postsurgical hypoparathyroidism was defined as PTH levels < 10 pg/mL following surgery (normal range = 10–65 pg/mL). In the case of PTH concentrations below the normal range for more than 12 months, hypoparathyroidism was considered permanent.

Postoperative complications were categorized according to the Clavien–Dindo classification. Transient and permanent hypoparathyroidism were considered as grade I (requiring electrolyte correction), unilateral recurrent laryngeal nerve injury and wound infection as grade II (needing corticosteroid therapy and antibiotic therapy, respectively), bilateral recurrent laryngeal nerve injury and cervical haematoma as grade IIIb (requiring surgical treatment under general anesthesia, tracheostomy and surgical revision of hemostasis, respectively).

### Statistical analysis

Statistical analyses were performed with MedCalc® 19.1.3. In univariate analysis, Fisher exact test or Chi-squared test were used for categorical variables and *t*-test for continuous variables. Multivariate analyses were performed to evaluate whether the BMI value (considered in this case as a continuous variable) had a significant role in the development of postoperative complications. For this purpose, a multivariate analysis was conducted for each individual complication (transient hypoparathyroidism, permanent hypoparathyroidism, cervical haematoma, unilateral RLN injury, bilateral RLN injury and wound infection), considering as independent variables, other than BMI, sex, age, operative time, thyroid weight and histological diagnosis. The results regarding the influence of BMI on postoperative complications were extrapolated from each multivariate analysis and represented in a single forest plot.

The difference in terms of operative times between the two groups examined was represented through a box plot.

*P* values < 0.05 were considered statistically significant.

## Results

A total of 1594 consecutive patients were submitted to thyroidectomy within the period analysed. Eight hundred and thirteen met the inclusion criteria: 31 (3.81%) were underweight, 361 (44.40%) normal-weight, 286 (35.18%) overweight, 94 (11.57%) obese and 41 (5.04%) morbidly obese. Six hundred and seventy-eight patients were included in Group A and 135 in Group B.

Age, sex and histopathological findings were comparable between the two groups, while the mean BMI was significantly higher in Group B than in Group A (34.04 ± 4.02 vs 24.03 ± 3.25, *P* < 0.001). These results are shown in Table 1, while in Table 2 are reported surgical outcomes and postoperative complications.

**Table 1:** Demographic data, BMI and histopathological findings

	Total (n = 813)	Group A (n = 678)	Group B (n = 135)	*P* value
Sex				
Male	238 (29.27%)	198 (29.20%)	40 (29.63%)	0.921
Female	575 (70.73%)	480 (70.80%)	95 (70.37%)	
Age (years, mean ± SD)	54.36 ± 14.11	53.99 ± 14.11	56.21 ± 14.01	0.094
BMI (kg/m^2^, mean ± SD)	25.69 ± 5.03	24.03 ± 3.25	34.04 ± 4.02	** < 0.001**
Thyroid weight (g, mean ± SD)	46.9 ± 51.41	44.77 ± 44.89	57.57 ± 75.49	**0.008**
Histological diagnosis				
Benign disease	506 (62.24%)	418 (61.65%)	88 (65.19%)	0.439
Malignancy	307 (37.76%)	260 (38.35%)	47 (34.81%)	

Statistically significant values are shown in bold

*SD* standard deviation, *BMI* body mass index

**Table 2:** Surgical outcomes and complications

	Total (n = 813)	Group A (n = 678)	Group B (n = 135)	*P* value
Operative time (minutes, mean ± SD)	87.66 ± 19.60	86.63 ± 19.20	92.81 ± 20.81	**0.001**
Postoperative stay (days, mean ± SD)	2.98 ± 1.53	2.94 ± 1.59	3.16 ± 1.19	0.123
Transient hypoparathyroidism	172 (21.16%)	147 (21.68%)	25 (18.52%)	0.411
Permanent hypoparathyroidism	48 (5.90%)	37 (5.46%)	11 (8.15%)	0.226
Cervical haematoma	9 (1.11%)	9 (1.33%)	0	0.369
Unilateral recurrent nerve injury	22 (2.71%)	19 (2.80%)	3 (2.22%)	1.000
Bilateral recurrent nerve injury	3 (0.37%)	3 (0.44%)	0	1.000
Wound infection	2 (0.25%)	2 (0.29%)	0	1.000

Statistically significant values are shown in bold

*SD* standard deviation

In Group A, the mean operative time was 86.63 ± 19.20 min and the mean postoperative stay was 2.94 ± 1.59 days. There were 19 (2.80%) unilateral recurrent laryngeal nerve lesions, 3 (0.44%) bilateral recurrent laryngeal nerve lesions, 9 (1.33%) cervical haematomas, 2 (0.29%) wound infections, 147 (21.68%) cases of transient hypoparathyroidism and 37 (5.46%) cases of permanent hypoparathyroidism.

In Group B, the mean operative time was 92.81 ± 20.81 min and the mean postoperative stay was 3.16 ± 1.19 days. There were 3 (2.22%) unilateral recurrent laryngeal nerve lesions, 25 (18.52%) cases of transient hypoparathyroidism and 11 (8.15%) cases of permanent hypoparathyroidism. No bilateral recurrent laryngeal nerve injury, cervical haematoma or wound infection occurred in this group.

According to the Clavien-Dindo classification, considering the whole sample, 211 (25.95%) patients were in grade I, 24 (2.95%) in grade II and 12 (1.48%) in grade IIIb.

Among patients in Group A, 176 (25.96%) were in grade I, 21 (3.10%) in grade II and 12 (1.77%) in grade IIIb.

Among patients in Group B, 35 (25.93%) were in grade I and 3 (2.22%) in grade II.

**Fig. 1:**
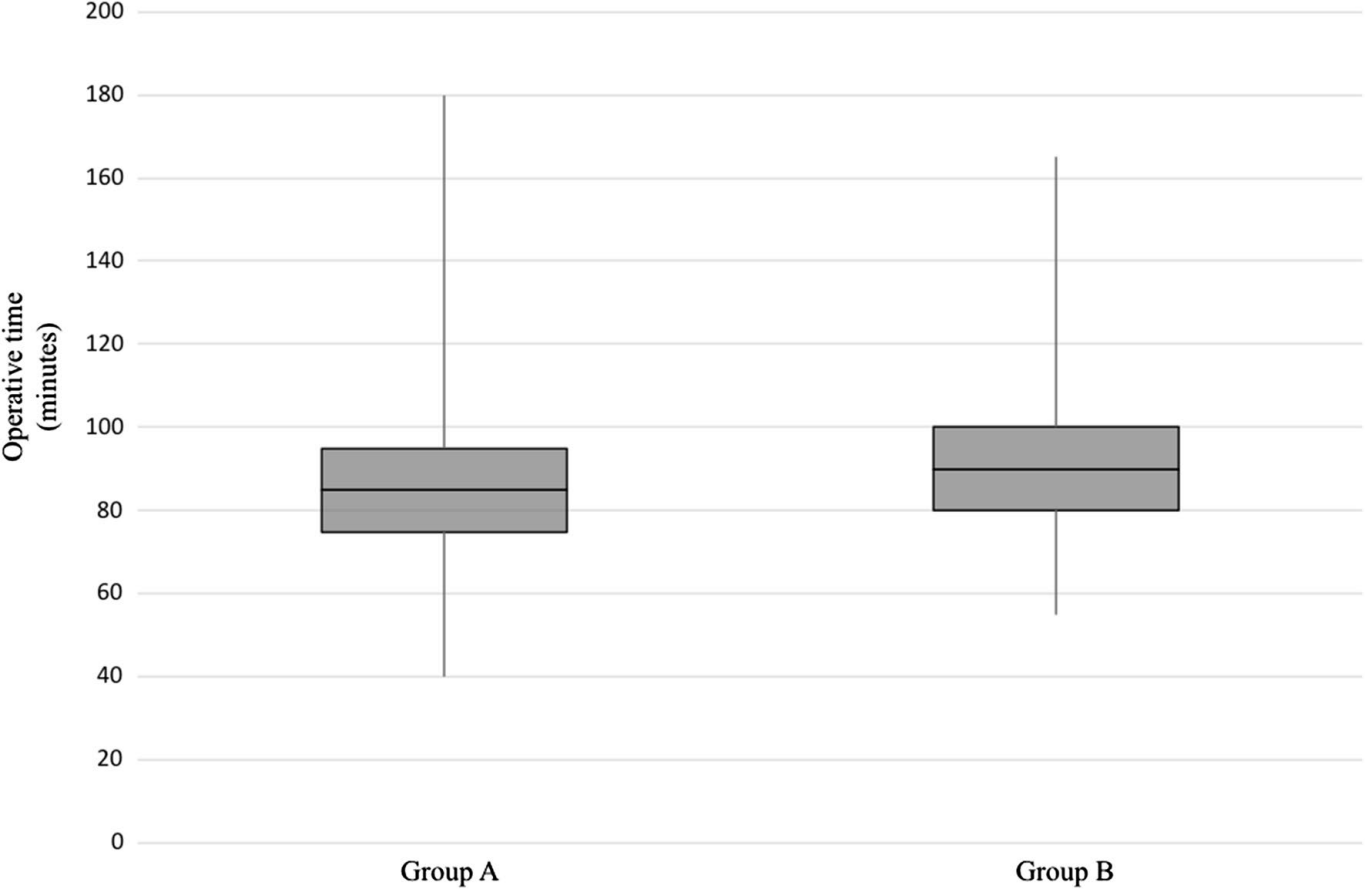
Box plot representing the difference in terms of operative times between the two groups

At univariate analysis, the comparison between the two groups, in terms of operative time and thyroid weight resulted in statistically significant results (*P* = 0.001, *P* = 0.008; respectively). These features were significantly higher in Group B than in Group A. The difference in operative times between the two groups was represented through a box plot in Fig. 1.

Differently, about postoperative stay and complications, no statistically significant difference was found between the two groups.

**Fig. 2:**
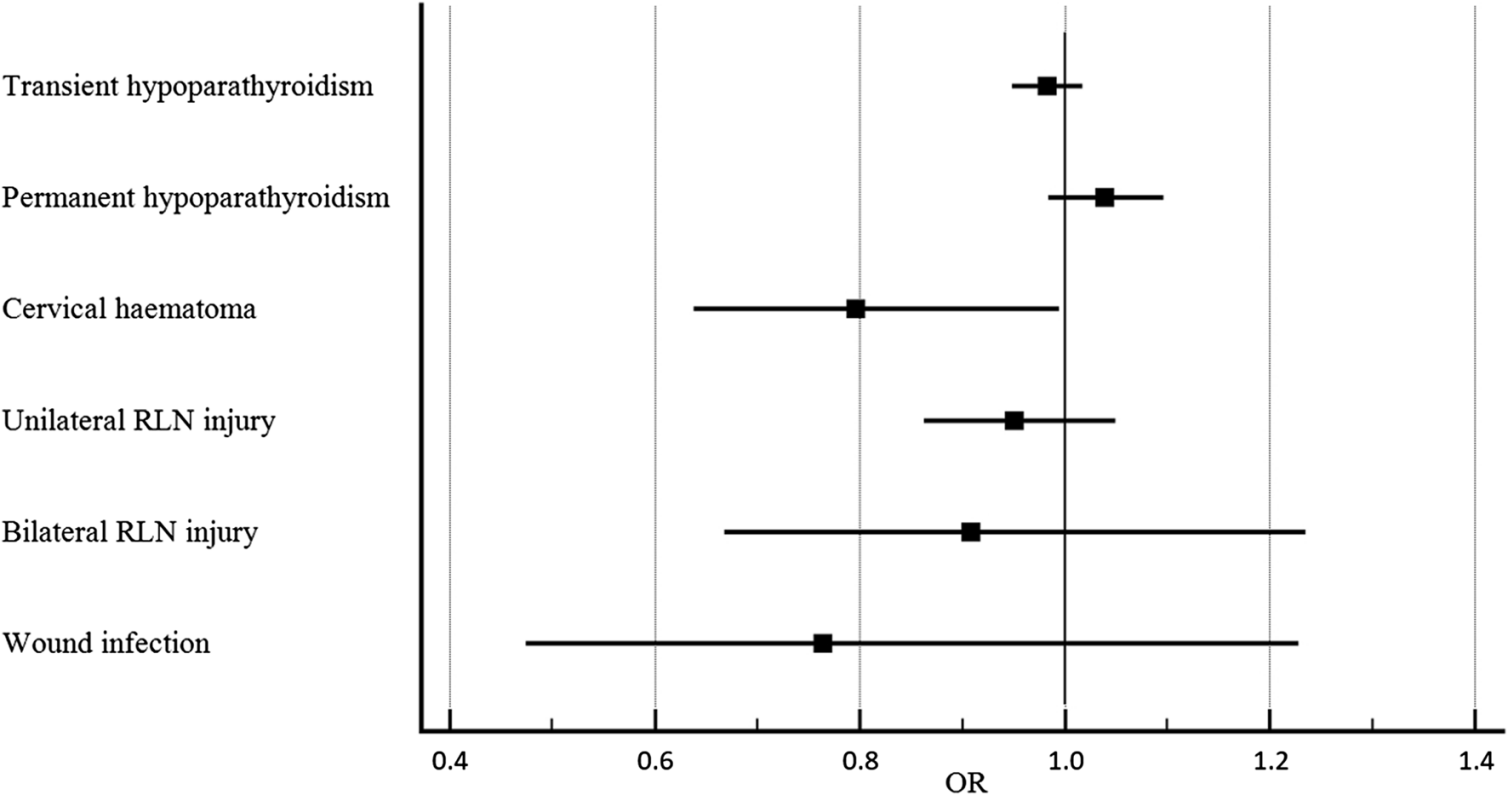
Forest plot representing the impact of BMI on the development of each individual postoperative complication

At multivariate analyses, only the development of cervical haematoma was statistically significantly correlated to the BMI value. As shown in Fig. 2, patients with high BMI had a lower risk of cervical haematoma (*P* = 0.045, OR 0.797, 95% CI 0.638–0.995).

## Discussion

Thyroidectomy is the most performed operation in endocrine surgery. It is widely utilized for the treatment of both benign and malignant thyroid diseases. Over 34,000 operations were performed in Italy during 2016 [20].

Although mortality due to this surgical procedure is negligible, morbidity remains a challenging problem, even in the most experienced hands. In this kind of surgery, morbidity is mainly represented by recurrent laryngeal nerve injury, hypoparathyroidism and cervical haematoma [21–25].

The correct positioning of the patient is considered fundamental to achieve the best exposure of the surgical site and therefore the best outcomes. It consists in hyperextension of the neck, which can be obtained through the interposition of a support between the operating table and patient’s shoulders. The wide and short neck of obese patients causes limited hyperextension, which results in a restricted operating field and, therefore, greater difficulty for the surgeon. It is precisely for this reason that many endocrine surgeons consider obese patients at greater risk of postoperative complications.

The aim of our study was to evaluate whether postoperative morbidity in obese patients undergoing thyroidectomy is really increased. In order to obtain a homogeneous sample and limit bias, only patients undergoing conventional open thyroidectomy alone were included in this work, while those submitted to minimally invasive video-assisted thyroidectomy (MIVAT) or who simultaneously underwent lateral and/or central neck dissection were excluded. Moreover, for the same purpose, only operations performed by the two most skilled endocrine surgeons of our Unit, with the same competence and experience in the field of thyroid surgery, were considered.

Also in our experience, as widely described in the literature, postoperative complications were not increased in obese patients. Furthermore, multivariate analyses even documented a lower risk of cervical haematoma in patients with high BMI.

As regards Clavien–Dindo classification, no patient was in grades IV and V. Moreover, it is important to note that no obese patient was in grade III.

About the postoperative stay, no statistically significant difference was found between the two groups.

As regards our result in terms of operative times, it was certainly influenced, at least in part, by the greater mean weight, and therefore size, of the thyroid gland in the group of obese patients. This difference in size may be due to a delayed diagnosis of thyroid disease in patients with elevated BMI, in whom cervical swellings deriving from thyroid nodules are more difficult to notice because of the high neck circumference. However, it is important to underline that, as in other studies, longer operative times had no clinical significance.

Most of the studies conducted so far on the correlation between elevated BMI and postoperative morbidity in the field of thyroid surgery confirm our findings [4, 10–13, 15]. Only three studies documented an increased occurrence of complications [5, 16, 17].

Buerba et al., who examined 18,825 patients undergoing thyroid surgery between 2005 and 2008, found that obese and morbidly obese patients had an increased risk of having at least one complication, especially wound complications. Moreover, they observed that morbid obesity was an independent predictor for urinary complications. However, it is important to underline that, in this study were not evaluated RLN injury and hypoparathyroidism, which are the two main complications of thyroidectomy [17].

Trésallet et al., analysing 1216 patients undergoing thyroidectomy for papillary thyroid carcinoma, observed no difference in terms of overall postoperative complications (including RLN injury, hypocalcaemia, bleeding requiring an emergency operative evacuation, abscess). However, they found that the risk of permanent complications, specifically RLN lesions, were greater in obese patients [5].

Jin et al. retrospectively reviewed 386 patients with papillary thyroid cancer who underwent total thyroidectomy and lateral neck dissection finding an increased occurrence of postoperative haematoma and wound infection in obese patients [16].

As regards operative times, as in our experience, an increase in patients with high BMI has generally been described [10, 12–14, 16, 17].

About the postoperative stay, only one study, conducted by Harari et al. on 443 patients undergoing thyroidectomy for papillary thyroid carcinoma, described an increase in patients with elevated BMI [4].

Our study has two main limitations. First of all, it is based on a retrospective analysis. The second limitation consists in the limited number of obese patients examined in relation to the low occurrence of some complications following thyroidectomy, specifically recurrent laryngeal nerve injury, cervical haematoma and wound infection. This last condition strongly hinders the achievement of a statistical power suitable for an accurate evaluation of these operative complications.

## Conclusion

This study showed that obesity, in the field of thyroid surgery, is not associated with any increase of postoperative complications. Thus, it is possible to conclude that thyroidectomy can be performed safely in obese patients.

Our result on operative times, certainly influenced, at least in part, by the greater mean size of the thyroid gland in patients with high BMI, had no clinical significance.

Future studies with larger populations, possibly multicenter, and meta-analyses are needed to better investigate this topic.

## Data Availability

The datasets used and/or analysed during the current study are available from the corresponding author on reasonable request.

## Abbreviations

WHO: World Health Organization
BMI: Body mass index
RLN: Recurrent laryngeal nerve
IONM: Intraoperative nerve monitoring
PTH: Parathyroid hormone
MIVAT: Minimally invasive video-assisted thyroidectomy
